# Supplementary material for: Transcriptome Analysis Reveals Vimentin-Induced Disruption of Cell–Cell Associations Augments Breast Cancer Cell Migration
Source: Cells. 2022 Dec 13;11(24):4035. doi: 10.3390/cells11244035 (PMC9776984; doi:10.3390/cells11244035)
Supplement: Supplementary file 1 [file cells-11-04035-s001.zip › DEGs and enrichment analysis.zip!/DEGs and enrichment analysis/WT_VIMvsVector KEGG/UP/WT_VIMvsVector_UP.pathway_map.zip!/WT_VIMvsVector_UP.pathway_map/src/hsa05165.html]

hsa05165


- hsa:3674

- Up regulated genes

ENSG00000005961(3.91653577454454)

- hsa:7015

- Up regulated genes

ENSG00000164362(2.48262040043416)

- hsa:1869

- Up regulated genes

ENSG00000101412(1.977544660594)

- hsa:890

- Up regulated genes

ENSG00000145386(1.72013468925064)

- hsa:23493

- Up regulated genes

ENSG00000135547(3.89723839640576)

- hsa:9368

- Up regulated genes

ENSG00000109062(1.72493546867558)

- hsa:7015

- Up regulated genes

ENSG00000164362(2.48262040043416)

- hsa:7283

- Up regulated genes

ENSG00000131462(2.14286842481096)

Close
